# Linking estrogen receptor β expression with inflammatory bowel disease activity

**DOI:** 10.18632/oncotarget.6217

**Published:** 2015-10-22

**Authors:** Marina Pierdominici, Angela Maselli, Barbara Varano, Cristiana Barbati, Paola Cesaro, Cristiano Spada, Angelo Zullo, Roberto Lorenzetti, Marco Rosati, Gabriella Rainaldi, Maria Rosaria Limiti, Luisa Guidi, Lucia Conti, Sandra Gessani

**Affiliations:** ^1^ Department of Cell Biology and Neurosciences, Istituto Superiore di Sanità, Rome, Italy; ^2^ Department of Therapeutic Research and Medicines Evaluation, Istituto Superiore di Sanità, Rome, Italy; ^3^ Department of Hematology, Oncology and Molecular Medicine, Istituto Superiore di Sanità, Rome, Italy; ^4^ Digestive Endoscopy Unit, Catholic University, Rome, Italy; ^5^ Gastroenterology and Digestive Endoscopy, Nuovo Regina Margherita Hospital, Rome, Italy; ^6^ Histopathology Complex Unit, Santo Spirito Hospital, Rome, Italy; ^7^ IBD Unit, Complesso Integrato Columbus, Catholic University, Rome, Italy

**Keywords:** estrogen receptors, inflammatory bowel disease, T lymphocytes, inflammation, cytokines, Immunology and Microbiology Section, Immune response, Immunity

## Abstract

Crohn disease (CD) and ulcerative colitis (UC) are chronic forms of inflammatory bowel disease (IBD) whose pathogenesis is only poorly understood. Estrogens have a complex role in inflammation and growing evidence suggests that these hormones may impact IBD pathogenesis. Here, we demonstrated a significant reduction (*p* < 0.05) of estrogen receptor (ER)β expression in peripheral blood T lymphocytes from CD/UC patients with active disease (*n* = 27) as compared to those in remission (*n* = 21) and healthy controls (*n* = 29). Accordingly, in a subgroup of CD/UC patients undergoing to anti-TNF-α therapy and responsive to treatment, ERβ expression was higher (*p* < 0.01) than that observed in not responsive patients and comparable to that of control subjects. Notably, ERβ expression was markedly decreased in colonic mucosa of CD/UC patients with active disease, reflecting the alterations observed in peripheral blood T cells. ERβ expression inversely correlated with interleukin (IL)-6 serum levels and exogenous exposure of both T lymphocytes and intestinal epithelial cells to this cytokine resulted in ERβ downregulation. These results demonstrate that the ER profile is altered in active IBD patients at both mucosal and systemic levels, at least in part due to IL-6 dysregulation, and highlight the potential exploitation of T cell-associated ERβ as a biomarker of endoscopic disease activity.

## INTRODUCTION

Crohn disease (CD) and ulcerative colitis (UC) are chronic forms of inflammatory bowel disease (IBD) arising from the combined effects of genetic susceptibility, environmental influences, intestinal dysbiosis, and innate and adaptive immune system dysregulation, leading to chronic inflammation and subsequent tissue damage [1, 2]. TNF-α, a molecule with a broad spectrum of activity, including costimulation of lymphocyte inflammatory functions and direct disruption of intestinal epithelial barrier integrity, has a well-established pathological role in IBD and has been successfully exploited as a target for therapeutic intervention [3]. However, subgroups of IBD patients fail to respond or lose response to this drug indicating that the immune networks in the inflamed mucosa are complex and subjected to multiple layers of regulation by still unrecognized factors [3].

Growing evidence from epidemiological and experimental studies suggests a role for estrogens, particularly 17β estradiol, in IBD pathogenesis [4-14]. Estrogens have been shown to finely regulate inflammation [15] and have been implicated in the modulation of several immune-mediated diseases [16, 17]. Most of their effects are mediated by two intracellular receptors, i.e, estrogen receptor (ER)α and ERβ, which function as ligand-activated nuclear transcription factors producing genomic effects [15]. ERs are expressed in different cell types including immune cells [18-21] and the presence of one ER subtype over the other may change estrogen effects, promoting or dampening inflammation [15].

ERβ is the predominant ER subtype in colon tissue, where it plays a fundamental role in growth, organization and maintenance of normal epithelial architecture [12, 22, 23]. Interestingly, reduced ERβ mRNA expression and increased gut permeability were found to precede the onset of colitis in mouse models of intestinal inflammation [12]. Additionally, ERβ knockout mice developed a clinically more severe colitis as compared to wild-type littermates [13]. In keeping with these findings, beneficial effects of ERβ agonists as well as estrogens have been reported in different models of chronic colitis [9-11, 14]. There is also evidence that estrogens can modulate disease severity in IBD patients [4-6]. However, ER expression in human IBD and its possible correlation with disease activity have been till now poorly investigated, with the exception of one study by Looijer-van Langen et al. [12] showing decreased ERβ mRNA levels in colonic biopsies from CD patients in relapse, but not in remission. Notably, UC and CD are associated with an increased risk of colorectal cancer (CRC) that appears to increase with the duration, severity and anatomic extent of colonic inflammation [24, 25]. In this respect, growing data support a role for estrogen/ER in the initiation and progression of CRC, and establish that protective effects of estrogen are exerted through ERβ [26-28].

In this study we report IBD-associated alterations of ER expression profile both in peripheral blood T lymphocytes and in colonic mucosa and their relationship with the clinical characteristics of the studied population. We also identified interleukin (IL)-6 as an unrecognized regulator of ER expression in circulating T cells and intestinal epithelium.

## RESULTS AND DISCUSSION

### ERβ expression is downregulated in peripheral blood T lymphocytes from active IBD patients

**Figure 1:**
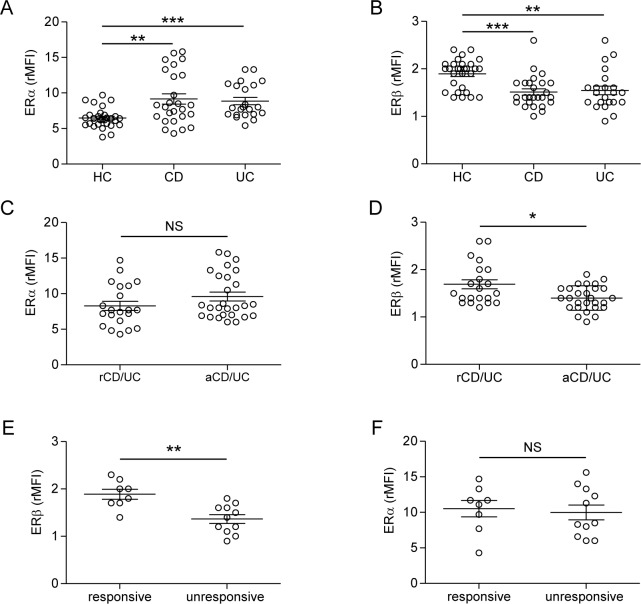
Intracellular ER expression in peripheral blood T lymphocytes from CD/UC patients **A.**-**D.** Intracellular ERα and ERβ expression levels evaluated by flow cytometry in T cells from CD/UC patients, considered as a whole (*n* = 48) or divided in patients in remission (*n* = 21) and those with active disease (*n* = 27) according to the endoscopic activity, and from healthy controls (HC; *n* = 29). **E.**, **F.** Intracellular ER expression evaluated in T cells from a subgroup of CD/UC patients in ongoing treatment with anti-TNF-α (*n* = 19), divided in responsive (*n* = 8) and unresponsive (*n* = 11) patients. Values of ER/isotype control mean fluorescence intensity ratio (rMFI) are reported (mean ± SEM is shown for each group). Statistical differences were calculated by the Mann-Whitney U test. *p < 0.05 ; **p < 0.01; ***p < 0.001. NS, nonsignificant; rCD/UC, CD/UC patients in endoscopic remission; aCD/UC, CD/UC patients with endoscopic activity.

We evaluated by flow cytometry the intracellular expression of ERα and ERβ in peripheral blood T lymphocytes from 48 patients with IBD (CD, *n* = 26 and UC, *n* = 22) and 29 age/sex matched healthy controls (HC). The demographic and clinical characteristics of IBD patients are summarized in Table 1. A significant increase of ERα and a concomitant decrease of ERβ expression were observed in T lymphocytes from IBD patients as compared to HC, whereas no differences were detected between CD and UC patients (Figure 1A and 1B). Similar results were obtained when purified CD4^+^ and CD8^+^ T cells were analyzed separately (data not shown). For both ERα and ERβ expression, no significant associations were found with the epidemiological data (sex, age) of the patient population. To estimate whether ER expression level reflects disease activity, the patient population was divided into 2 groups according to the endoscopic activity at the time of sampling, i.e., patients with active disease and those in remission (see Materials and Methods and Table 1 for details). Although ERα expression was not significantly different in T cells from patients in remission and those with active disease (Figure 1C), a significantly lower expression of ERβ was found in T cells from CD/UC patients with active disease as compared to those in remission (Figure 1D).

**Table 1:** Demographic and clinical characteristics of the study sample

	CD	UC
Patients, n of patients	26/48	22/48
Age (years), median (range)	45 (19-70)	44 (19-68)
Women/men	11/15	10/12
Endoscopic remission^a^, n of patients (%)	12 (46)	9 (41)
Current drug therapy, n of patients (%)		
5-aminosalicylic acid	8 (31)	10 (45)
Systemic corticosteroid	2 (7)	2 (9)
Anti-TNFα (Infliximab/Adalimumab)	12 (46)	7 (32)

a CD: Crohn's Disease Endoscopic Index of Severity (CDEIS, remission when score < 3); post-operative CD: Rutgeerts score (remission when score = 0); UC: Mayo endoscopic sub-score (remission when score = 0).

Although ERs have been shown to finely regulate inflammation [15], this is the first demonstration of a specific alteration of ER profile in IBD. The current diagnosis and management of IBD is based on clinical and endoscopic criteria [29]. More specifically, as routine clinical assessment is often inaccurate with respect to endoscopic activity [30], colonoscopy represents the gold standard technique for the evaluation of disease severity. However, due to the complexity and invasiveness of this practice, there is a pressing need for new non-invasive biomarkers to improve disease activity detection, in order to better determine prognosis and to monitor drug response. In this regard, the strong association between lymphocyte ERβ levels and endoscopic disease activity observed in our study points to this receptor as a potential prognostic biomarker for IBD.

Interestingly, blood T lymphocytes from a subgroup of CD/UC patients in ongoing treatment with anti-TNF-α (infliximab or adalimumab: 12/26 CD and 7/22 UC) showed significantly different expression of ERβ according to drug response, as monitored by the endoscopic activity. Specifically, responsive patients (*n* = 8) expressed higher levels of ERβ as compared to unresponsive patients (*n* = 11) (Figure 1E). The expression of ERα was found to be not significantly different between these 2 groups of patients (Figure 1F). As response to therapy has been established on the basis of disease remission at the endoscopic level, our findings further strengthen the role of T cell-associated ERβ as a systemic marker of intestinal disease activity. Additionally, the association found between anti-TNF-α response and normal ERβ levels in blood T lymphocytes suggests that ERβ may represent a candidate predictive marker to assess responsiveness to biological therapy. However, longitudinal studies including subjects analyzed before and after the initiation of anti-TNF-α therapy are needed to provide conclusive evidence for a strict association between anti-TNF-α response and ERβ rescue.

### ERβ dysregulation in peripheral blood reflects that observed in intestinal mucosa

**Figure 2:**
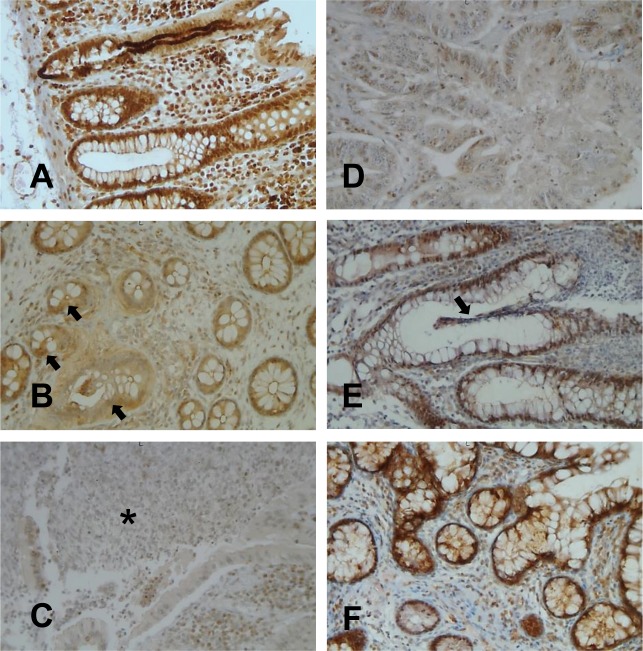
ERβ expression in colonic mucosa from CD/UC patients Light microscopic micrographs of typical ERβ expression (brown staining) in normal colonic mucosa **A.**, colonic mucosa from UC **B.** or CD **C.** patients in the active phase of disease, colonic mucosa from UC **E.** or CD **F.** patients in remission, and colon adenocarcinoma **D.**. Arrays indicate the disrupted or regenerating epithelium typical of UC mucosa in the active phase of disease **B.** and in remission **E.**, respectively. The asterisk indicates the typical fistula distinguishing CD mucosa **C.**. One representative image of each group (UC, *n* = 10; CD, *n* = 9; HC, *n* = 10; colon adenocarcinoma patients, *n* = 5) is shown (original magnifications x20).

It has been previously shown that the accumulation of ERβ transcripts is decreased in intestinal mucosa samples from IBD patients as compared to controls [12]. To assess the expression of ERβ protein in intestinal mucosa of IBD patients and to evaluate whether this expression reflects that observed in the blood, ERβ was also analyzed in whole colonic mucosa from 19 out of 48 CD/UC patients and 10 age/sex matched HC. As shown in Figure 2A, ERβ was highly expressed in normal colonic mucosa, where a positive staining could be observed in both epithelial cells and lamina propria lymphocytes. Interestingly, a consistent reduction of ERβ expression was found in intestinal mucosa from CD/UC subjects with active disease (Figure 2B and 2C) but not from those in remission (Figure 2E and 2F). In particular, a reduction of both epithelial cell- and lymphocyte-associated ERβ levels could be appreciated in inflamed mucosa (Figure 2B and 2C). Consistent with previous reports [26, 31, 32], neoplastic mucosa explanted from patients with colon adenocarcinoma (*n* = 5) displayed very low levels of ERβ expression (Figure 2D).

These results provide the first evidence that, in human colon, ERβ is markedly reduced in both CD and UC patients and the extent of its expression reflects disease activity. Of note, ERβ down-regulation in the colon tissue parallels that observed in blood T lymphocytes, further strengthening the role of T cell-associated ERβ as a systemic marker of intestinal disease activity. Moreover, the finding that colonic ERβ expression inversely correlates with cancer development/progression [26, 31, 32], together with our results, point to ERβ as a major regulator of intestinal epithelium integrity also in the human system and suggest a role for this receptor in maintaining homeostasis. Of note, IBD represent a risk factor for CRC development [24, 25], and inflammation-associated early ERβ down-regulation might be one of the factors linking chronic intestinal diseases to neoplastic transformation.

### IL-6 plasma levels inversely correlate with ERβ expression and exogenous exposure of both lymphocytes and intestinal epithelial cells to recombinant IL-6 results in ERβ downregulation

The unbalanced expression of ERα and ERβ, exhibiting pro- *versus* anti-inflammatory features, respectively, suggests that a switch toward ER-mediated inflammatory responses occurs in CD/UC patients. Whether the alterations of ER and ER-induced responses contribute to establish or are a consequence of the chronic inflammatory process observed in these subjects is currently unknown. The finding that a similar ER regulation occurred in both CD and UC, which exhibit different localization, endoscopic findings and histological features, would support the latter hypothesis.

**Figure 3:**
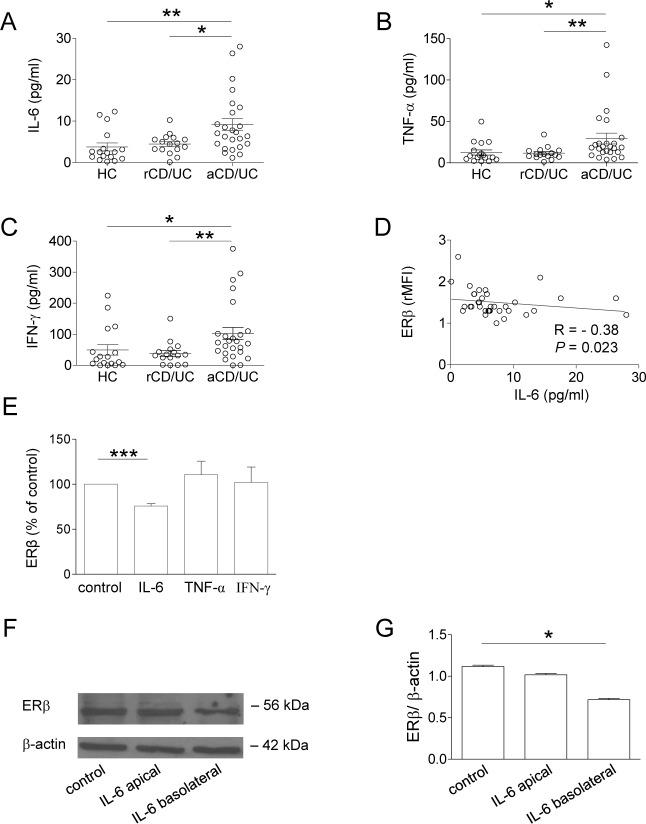
Plasma cytokine profile of CD/UC patients and effect of IL-6 on ERβ expression in peripheral blood T lymphocytes and intestinal epithelial cells **A.**-**C.** Cytokine plasma levels from 41 CD/UC patients (*n* = 25 with active disease, *n* = 16 in remission) and 17 HC. Mean ± SEM is shown for each group. **D.** Correlation between plasma levels of IL-6 and ERβ expression (shown as ERβ/isotype control mean fluorescence intensity ratio, rMFI) in T lymphocytes from CD/UC patients determined by the Spearman's rank correlation test. R, Spearman's rho. **E.** Intracellular ERβ expression evaluated by flow cytometry in T lymphocytes from HC exposed for 72 h to the indicated cytokines. Data are expressed as percentage relative to untreated cells (mean ± SEM of 5 independent experiments is shown). **F.**, **G.** Western blot analysis of ERβ expression in differentiated Caco-2 cells stimulated with IL-6 at either apical or basolateral side. Data from one representative experiment out of three are shown **F.**. Densitometry analysis of protein levels relative to β-actin is also shown **G.**. Values are expressed as mean ± SEM. Statistical differences were calculated by the Mann-Whitney U test. *p < 0.05 ; **p < 0.01; ***p < 0.001. rCD/UC, CD/UC patients in endoscopic remission; aCD/UC, CD/UC patients with endoscopic activity.

The analysis of soluble immune mediators in plasma samples from CD/UC patients revealed significantly higher levels of the pro-inflammatory cytokines IL-6, TNF-α and IFN-γ in subjects with active disease as compared to those in remission (Figure 3A-3C). Interestingly, a significant inverse correlation was found between ERβ expression and plasma levels of IL-6 (Figure 3D) but not those of TNF-α and IFN-γ (R = −0.10, *p* = 0.58, and R = −0.07, *p* = 0.69, respectively, data not shown). No correlation was found between ERα expression and plasma cytokine levels (data not shown).

Based on these results, the effect of exogenous administration of IL-6 on ERβ expression was investigated. As shown in Figure 3E, exposure of control blood T lymphocytes to IL-6 significantly reduced ERβ expression, whereas TNF-α and IFN-γ did not exert any effect. Conversely, ERα levels were not significantly modulated by these cytokines (data not shown). To assess whether intestinal ERβ expression could be regulated as a result of inflammatory cytokine stimulation, Caco-2 cell-derived normal epithelium was exposed to IL-6 at either apical or basolateral sides. Consistent with the results obtained in T cells, IL-6 was found to downregulate ERβ expression in intestinal epithelial cells. Notably, this effect was observed when IL-6 was added to the basolateral but not to the apical side (Figure 3F and 3G), thus resembling the cross-talk between intestinal epithelium and lamina propria immune cells occurring *in vivo* [33].

Although the mechanism(s) underlying ERβ regulation remain to be further elucidated, our results show that the pro-inflammatory cytokine IL-6, whose levels we found increased in serum from IBD patients with active disease, contributes to regulate ER expression in T lymphocytes and in *in vitro*-generated intestinal epithelium. IL-6 has been recently reported to negatively regulate ERβ expression in ovarian cancer cell lines [34]. To the best of our knowledge, this is the first demonstration that this cytokine can regulate ERβ expression in intestinal epithelium and blood T cells, suggesting that the inflammatory microenvironment of IBD patients plays a role in controlling the ERα/ERβ balance. In keeping with this hypothesis, the potential of IL-6 targeting as a therapeutic strategy in IBD is under investigation [35].

A deeper characterization of factors and mechanisms controlling ER expression and function may increase our knowledge on the pathogenesis of IBD and open new perspectives for the comprehension and management of the disease. Further analysis using ERα and ERβ selective agonists will allow to more deeply investigate the ER-mediated responses elicited in chronic inflammatory conditions, and to study the potential role of ER as therapeutic target. Reliable biomarkers of disease activity as well as of response to therapy are not yet available in IBD. Thus, identifying new factors/mechanisms that are dysregulated in IBD patients may open new perspectives for the comprehension of disease pathogenesis and clinical management. Overall, our results indicate that a better understanding of the role of ERs may advance our knowledge of these complex diseases, and lead to novel therapeutic options.

## MATERIALS AND METHODS

### Ethics statement

Investigation has been conducted in accordance with the ethical standards and according to the Declaration of Helsinki and according to national and international guidelines and has been approved by the authors' institutional review board. All the subjects included were provided with complete information about the study and asked to sign an informed consent.

### Patients and biological samples

Biological samples were obtained from patients with documented CD (*n* = 26) or UC (*n* = 22) attending to the Digestive Endoscopy Unit (Catholic University, Rome, Italy), the Gastroenterology and Digestive Endoscopy (Nuovo Regina Margherita Hospital, Rome, Italy), and the IBD Unit (Complesso Integrato Columbus, Catholic University, Rome, Italy). Endoscopic biopsies from 5 patients with colon adenocarcinoma were obtained from the Histopathology Complex Unit (Santo Spirito Hospital, Rome, Italy). Twenty-nine healthy controls (HC) matched for age and sex made up a control group. The exclusion criteria were: clinical evidence of active infection, recent (within 14 days) use of antibiotics, pregnancy, hormone-based therapy, and treatment with corticosteroids (methylprednisolone or equivalents) at doses > 20 mg/day. Patients undergoing anti-TNF-α therapy were analyzed after at least 6 months from the initiation of therapy and blood ER expression was determined the week before the next anti-TNF-α administration. Endoscopic results were assessed according to the Crohn's Disease Endoscopic Index of Severity (CDEIS, remission when score < 3) [36] for CD, the Rutgeerts score (remission when score = 0) [37] for post-operative CD, and the Mayo endoscopic sub-score (remission when score = 0) [38] for UC. Blood samples were drawn at the time of obtaining peripheral vein access for the endoscopic procedure. Plasma were frozen soon after collection and stored at −80°C until used. Endoscopic biopsies were taken from colon/ileum tissue of IBD patients, whereas surgical specimens of colonic mucosa, from both neoplastic and adjacent (> 5 cm distal) macroscopically non-neoplastic regions, were obtained from subjects with colon adenocarcinoma. Intestinal mucosa was fixed in 10% formalin and stored for further processing.

### Isolation of peripheral blood mononuclear cells and cell culture conditions

Peripheral blood mononuclear cells were isolated by Ficoll-Hypaque density-gradient centrifugation. Untouched T cells were subsequently separated using the Pan T Cell isolation Kit II (Miltenyi Biotec, Bergisch-Gladbach, Germany). The purity of recovered cells, assessed by flow cytometer, was ≥ 97%. For ER analysis, cells were processed soon after isolation. For cytokine stimulation, T cells isolated from HC were seeded in RPMI-1640 medium without phenol red (GIBCO BRL, Grand Island, NY, USA) supplemented with 10% charcoal-stripped fetal bovine serum (FBS, Euroclone, Pero, Milan, Italy), 2mM glutamine (Sigma, St. Louis, MO, USA) 50 μg/ml gentamycin (Sigma), and exposed for 72 hours to IL-6 or TNF-α (Peprotech, Rocky Hill, NJ, USA; 50 ng/ml) or IFN-γ (Peprotech, 500 IU/ml) before ER analysis. The 72 hour time point as well as the concentrations used were chosen on the basis of preliminary time course and dose-response experiments showing that these conditions were those at which the highest changes of ER expression could be detected.

### Flow cytometry

Intracellular phenotyping of T cells was performed by flow cytometry as previously described [21]. FITC-conjugated rabbit anti-human ERα antibody (Ab, clone MC-20) or mouse anti-human ERβ (clone 1531) Ab, both from Santa Cruz Biotechnology (Santa Cruz, CA, USA), were used. Equal amounts of appropriate isotype controls (Santa Cruz Biotechnology) were used. Anti-human ERβ Ab was visualized by fluorescein isothiocyanate-conjugated F(ab')2 fragment secondary Ab (Abcam, Cambridge, UK). Allophycocyanin conjugated anti-CD3, phycoerythrin conjugated anti-CD4, peridinin chlorophyll protein-conjugated anti-CD8 monoclonal Abs (all from BD Biosciences, San Jose, CA, USA) were also used to identify ERα and ERβ expression in lymphocyte subsets. Acquisition was performed on a FACSCalibur cytometer (BD Biosciences) and 50,000 events per sample were run. Data were analyzed using the Cell Quest Pro (BD Biosciences) software.

### Quantification of plasma cytokine/chemokine levels

The analysis of cytokine content in plasma samples of randomly selected HC and IBD patients was performed by using the Bio-Plex Pro Human Cytokine 27-plex Assay (Bio-Rad Laboratories, Hercules, CA, USA) according to the manufacturer's instructions. This assay was able to concomitantly detect the following cytokines, chemokines and growth factors: IL-1β, IL-1ra, IL-2, IL-4, IL-5, IL-6, IL-7, IL-8, IL-9, IL-10, IL-12 (p70), IL-13, IL-15, IL-17, Basic FGF, Eotaxin, G-CSF, GM-CSF, IFN-γ, IP-10, MCP-1 (MCAF), MIP-1α, MIP-1β, PDGF-BB, RANTES, TNF-α, VEGF. The assay was performed at the Facility for Complex Protein Mixture (CPM) Analysis (Istituto Superiore di Sanità, Rome, Italy).

### Immunohistochemistry analysis

Intestinal mucosa sections were mounted on to superfrost microscope slides and immunostaining of ERβ was performed. Mouse anti-human ERβ Ab (clone 14C8, Abcam) was applied at a dilution of 1:200 for 60 minutes at 37°C. Antigen retrieval was performed with Cell Conditioning (CC1, Ventana Medical Systems, Illkirch, France) for 60 minutes at 98°C. The Ab reactions were revealed using the ultraView Universal DAB (Ventana Medical Systems). ERβ staining of neoplastic mucosa from subjects with colon adenocarcinoma was also performed, whereas adjacent normal mucosa (> 5 cm distal from the neoplastic lesion) was used as a positive control. Sections incubated after the omission of the primary Ab were used as negative controls. The sections were counterstained with haematoxylin, dehydrated and mounted on glass coverslips. All samples were observed and photographed with a Zeiss Axiovert microscope.

### Caco-2 cell based model of intestinal epithelium and western blot analysis of ERβ expression

Polarized human intestinal epithelium was obtained by culturing Caco-2 cells (ATCC #HTB-37) on polycarbonate-coated trans-well chambers (8×10^4^ cell/cm^2^) in high glucose Dulbecco's Modified Eagle Medium (DMEM) plus 10% FBS and nonessential amino acids for 21 days as previously described [39]. Trans epithelial electrical resistance was monitored throughout the differentiation period. Differentiated cell cultures were then stimulated for 24 hours with recombinant IL-6 (50 ng/ml) at either apical or basolateral sides, then harvested and lysed in RIPA buffer. Intracellular ERβ expression analysis was performed by western blot as previously described [21], using a mouse anti-human ERβ Ab (clone 1531, Santa Cruz Biotechnology). To ensure the presence of equal amounts of protein, the membranes were re-probed with a rabbit anti-human β-actin Ab (Sigma).

### Statistics

The Mann-Whitney U test was used for comparisons between different groups. Correlations were evaluated using Spearman's rank correlation test. Linear regression analysis was performed to display a best fit line to the data. Statistical analyses were performed using GraphPad Prism 5 software. All tests were 2-sided and a *p* value < 0.05 was considered statistically significant.
